# E6AP promotes prostate cancer by reducing p27 expression

**DOI:** 10.18632/oncotarget.17224

**Published:** 2017-04-19

**Authors:** Dinesh Raghu, Piotr Jan Paul, Twishi Gulati, Siddhartha Deb, Christine Khoo, Andrea Russo, Enzo Gallo, Giovanni Blandino, Ai-Leen Chan, Elena Takano, Shahneen K. Sandhu, Stephen B. Fox, Scott Williams, Sue Haupt, Cristina Gamell, Ygal Haupt

**Affiliations:** ^1^ The Sir Peter MacCallum Department of Oncology, The University of Melbourne, Melbourne, Victoria, Australia; ^2^ Tumor Suppression Laboratory, Peter MacCallum Cancer Centre, Melbourne, Victoria, Australia; ^3^ Anatpath Services Pty Ltd, Gardenvale, Victoria, Australia; ^4^ Department of Pathology, Peter MacCallum Cancer Centre, Melbourne, Victoria, Australia; ^5^ Department of Surgical Pathology, Regina Elena Cancer Institute, Rome, Italy; ^6^ Oncogenomic and Epigenetic Unit, Italian National Cancer Institute, Rome, Italy; ^7^ Division of Cancer Medicine, Peter MacCallum Cancer Centre, Melbourne, Victoria, Australia; ^8^ Division of Radiation Oncology and Cancer Imaging, Peter MacCallum Cancer Centre, Melbourne, Victoria, Australia; ^9^ Department of Biochemistry and Molecular Biology, Monash University, Melbourne, Victoria, Australia; ^10^ Department of Pathology, The University of Melbourne, Melbourne, Victoria, Australia; ^11^ Current address: Australian Regenerative Medicine Institute, Monash University, Melbourne, Victoria, Australia

**Keywords:** prostate cancer, E6AP, p27, E2F1, tumor suppression

## Abstract

Prostate cancer (PC) is the most common cancer in men. Elevated levels of E3 ligase, E6-Associated Protein (E6AP) were previously linked to PC, consistent with increased protein expression in a subset of PC patients. In cancers, irregular E3 ligase activity drives proteasomal degradation of tumor suppressor proteins. Accordingly, E3 ligase inhibitors define a rational therapy to restore tumor suppression. The relevant tumor suppressors targeted by E6AP in PC are yet to be fully identified. In this study we show that p27, a key cell cycle regulator, is a target of E6AP in PC. Down regulation of E6AP increases p27 expression and enhances its nuclear accumulation in PC. We demonstrate that E6AP regulates p27 expression by inhibiting its transcription in an E2F1-dependent manner. Concomitant knockdown of E6AP and p27 partially restores PC cell growth, supporting the contribution of p27 to the overall effect of E6AP on prostate tumorigenesis. Overall, we unravelled the E6AP-p27 axis as a new promoter of PC, exposing an attractive target for therapy through the restoration of tumor suppression.

## INTRODUCTION

Prostate cancer (PC) is one of the leading causes of cancer death among men [1, 2]. When not cured by local therapy, patients commonly progress to castration resistant PC (CRPC), which is a lethal form of the disease with limited treatment options. Promising PC treatment through tumor suppression restoration is emerging using proteasome inhibitors. To date, the inhibitors available (eg. Bortezomib [1, 2]) lack specificity and their poorly-defined, broad-spectrum impact, is often associated with significant side effects. Defining critical E3 ligases and their substrates along the PC-driving proteasomal degradation pathways will facilitate the development of more effective and selective therapies.

E6AP (E6–Associated Protein) is the prototype of the E3 ligases characterised by a C-terminal HECT domain [3]. E6AP was initially discovered through its important role in high-risk Human Papilloma Virus (HPV)-mediated cancer, where it promotes p53 degradation [4]. However in recent years, its role in HPV-independent cancers has emerged [5–7, 24]. This includes the involvement of E6AP in the promotion of PC growth [8–10]. In response to E6AP overexpression, mice prostate glands are enlarged and exhibit prostatic intraepithelial neoplasia (PIN) [10]; while correspondingly in E6AP-deficient mice prostate development is reduced [11]. We recently showed that knockdown of E6AP attenuates the growth of PC cells *in vitro* and *in vivo*, and that this effect is mediated, in part, through an E6AP-target we previously identified [12]: the tumor suppressor Promyelocytic Leukaemia protein (PML). Consistently, in a subset of patients with localised PC, the combination of high E6AP/low PML expression levels is a prognostic marker for PC-associated death [8]. However, a subset of PC patients with high E6AP expression are not associated with low PML levels, predicting that other tumor suppressors are targeted by E6AP in these patients.

A key tumor suppressor whose expression is down regulated in many cancers, including PC, is p27 a pivotal cell cycle regulator (reviewed in [13]). P27, also known as KIP1, is encoded by the *CDKN1B* gene and it regulates cell cycle progression from quiescent to G1 and from G1 to S phase by inhibiting cyclin-dependent kinases (CDKs)/cyclin complexes [14, 15]. Both reduction in p27 expression levels and a decrease in its nuclear accumulation have been associated with PC pathogenesis [16, 17]. Consistently, loss of p27 in the PTEN+/− PC mouse model correlates with increased risk of recurrent disease, tumor burden, cell proliferation and invasiveness [18]. Moreover, downregulation of both CDK inhibitors p21 and p27 in a DU145 xenograft mouse model results in more aggressive tumors [19]. Interestingly, deletion or mutations of the *CDKN1B* gene are rare in cancers. This suggests that p27 is largely deregulated in cancer at the expression level and by altered subcellular localization (reviewed in [13]).

In this study, we have demonstrated a new link between E6AP and p27 in PC. Our data demonstrates this link in human PC samples, in a xenograft mouse model and in cultured PC cell lines. We identified the levels at which E6AP regulates p27 and propose a mechanistic explanation for its loss in PC. Importantly, our study exposes a novel E6AP-p27 axis contributing to PC pathogenesis and exposes a new therapeutic opportunity.

## RESULTS

### High E6AP expression correlates with low p27 protein levels in PC

**Figure 1:**
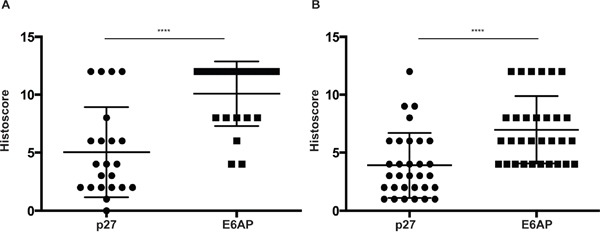
Expression levels of E6AP and p27 inversely correlate in PC TMA1 (n=47) **(A)** and TMA2 (n=117) **(B)** from PC patients were stained for E6AP and p27 and scored using a combined measure of the proportion of stained cells and the staining intensity. Intensity of staining ranged from 0 to 3 and the proportion of cells stained was based on a scale of 0 to 4 (0 to >80% respectively). The multiplied product of nuclear intensity and the proportion of stained cells was plotted as histoscore with S.D. *P-values* were calculated by unpaired student *t*-test (****p<0.0001).

To investigate the role of E6AP-p27 axis in a clinical setting, we analysed E6AP and p27 protein levels by immunohistochemistry in two cohorts of PC patients using tissue micro-arrays (TMAs). The two independent TMAs represent patients from different stages of the disease; TMA1 contains biopsies sampled from 47 PC patients with Gleason score 6-9 and TMA2 contains 117 biopsies from PC patients with Gleason score 3-7. The product of the proportion (scale: 0-4) of positive cancer cells and their nuclear staining intensity (scale: 0-3) was then derived to create a histoscore of 0-12 for each sample. The analysis of the two TMA cohorts revealed that patients with high levels of E6AP (mean histoscore ~10 for TMA1 and 7 for TMA2) predominantly express low levels of p27 (mean histoscore 4) (Figure 1). Markedly, the proportion of patients with a high E6AP/low p27 expression pattern was greater in the TMA representing PC patients with the most elevated Gleason scores (46% of patients, TMA1, Figure 1A) than in the TMA representing cancers with lower Gleason score (26% of patients, TMA2, Figure 1B), suggesting that the high E6AP/low p27 correlation is stronger in late stages of the disease. The inverse nature of the correlation between the levels of E6AP and p27 was also demonstrated in the reverse dynamic (low E6AP/high p27). The analysis of TMA2 showed that most of the patients with high p27 levels (mean histoscore 7, 47% of patients) presented low levels of E6AP (Supplementary Figure 1A). These results demonstrate a strong inverse correlation between E6AP and p27 expression levels and identify a subset of patients with high E6AP/low p27 expression pattern (32%).

### Downregulation of E6AP restores p27 expression in PC

**Figure 2:**
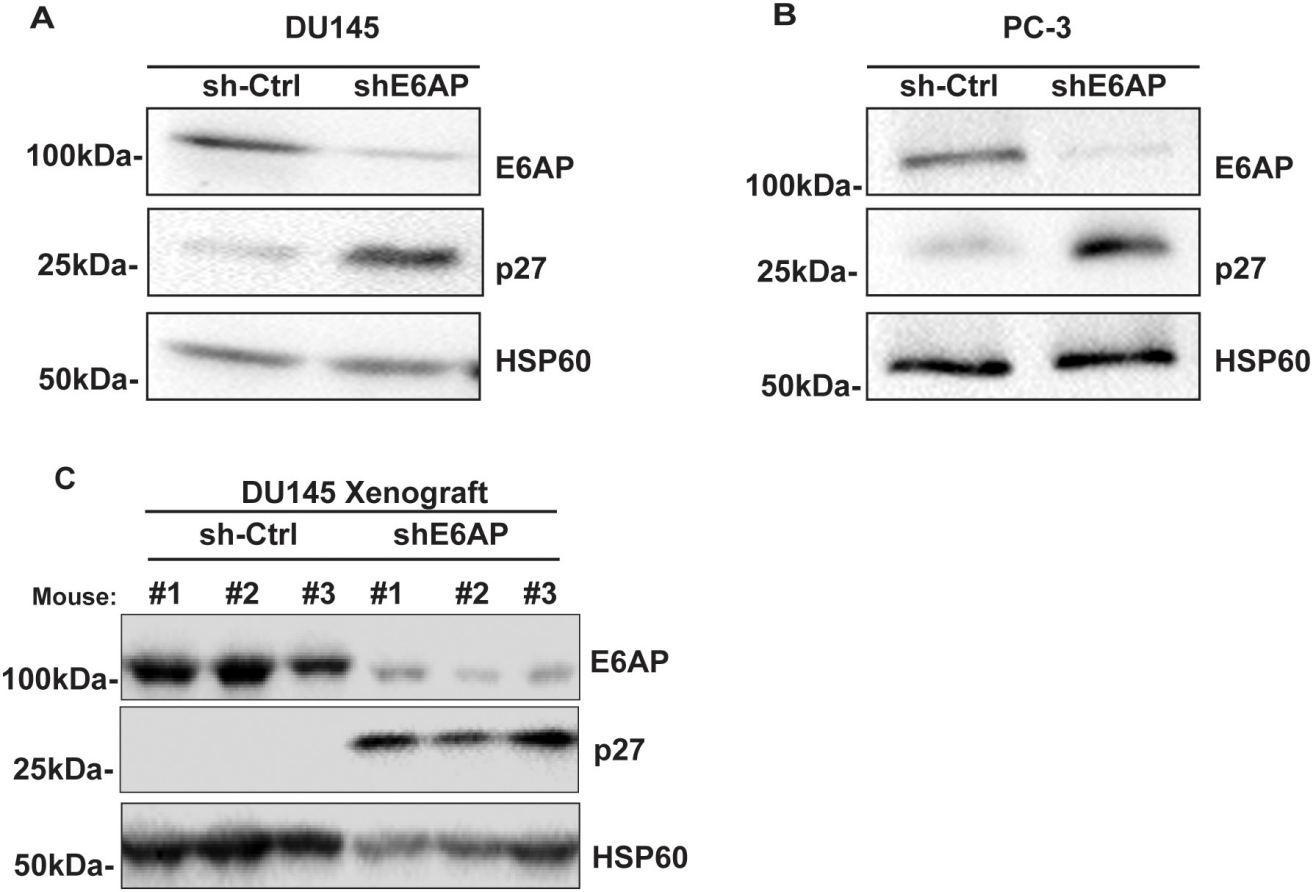
Knockdown of E6AP restores p27 protein levels *in vitro* and *in vivo* sh-Ctrl and shE6AP transduced DU145 **(A)** and PC-3 **(B)** cell lines were treated with 0.2μg/mL Dox for 96hrs. Samples were analyzed for the expression of E6AP, p27 and HSP60 (loading control) by western blotting. Representative images of three independent experiments are presented here. **(C)** sh-Ctrl and shE6AP transduced DU145 cells were injected subcutaneously in NSG mice and treated with Dox as detailed in Material and Methods. Tumor samples were analyzed by western blotting for the expression of E6AP, p27 and HSP60 (loading control).

We recently demonstrated that downregulation of E6AP attenuates growth of PC cells by restoring the expression of the tumor suppressor PML [9]. There is however a proportion of PC patients (12%) that express high levels of E6AP without the corresponding low levels of PML [8]. This argues that tumor suppressor(s) other than PML are targeted by E6AP in PC. Mishra et al [20] have previously shown that E6AP negatively regulates the expression levels of the tumor suppressor p27 in neuronal cells. Our identification of the inverse correlation between high E6AP and low p27 protein levels in a subset of PC patients (Figure 1) suggested a functional relationship whereby E6AP negatively targets p27 in PC cells. To test this hypothesis, we measured the effect of E6AP knockdown on p27 expression *in vitro*. DU145 and PC-3 PC cells were transduced with a doxycycline (Dox)-inducible conditional GFP-tagged lentiviral construct with a short hairpin against E6AP (shE6AP) or a hairpin Control (sh-Ctrl). Knockdown of E6AP resulted in elevated levels of p27 in DU145 (Figure 2A) and in PC-3 (Figure 2B). This increase in p27 expression was also observed using two additional shRNAs targeting E6AP (Supplementary Figure 2).

We next evaluated the effect of E6AP knockdown on p27 levels in an *in vivo* setting using a DU145 xenograft model. We have recently used this model to demonstrate that knockdown of E6AP in immunocompromised NOD/SCID/IL2rγ^null^ (NSG) mice results in tumor growth attenuation and extended survival [9]. Western blot analysis of tumors collected at ethical endpoint revealed that E6AP knockdown restored p27 protein expression compared with sh-Ctrl (Figure 2C). Taken together, these results show that knockdown of E6AP partially restores p27 expression in PC cells *in vitro* and *in vivo*, suggesting that E6AP negatively regulates p27 protein expression in PC cells.

### Downregulation of E6AP results in an increase in nuclear p27 protein levels

**Figure 3:**
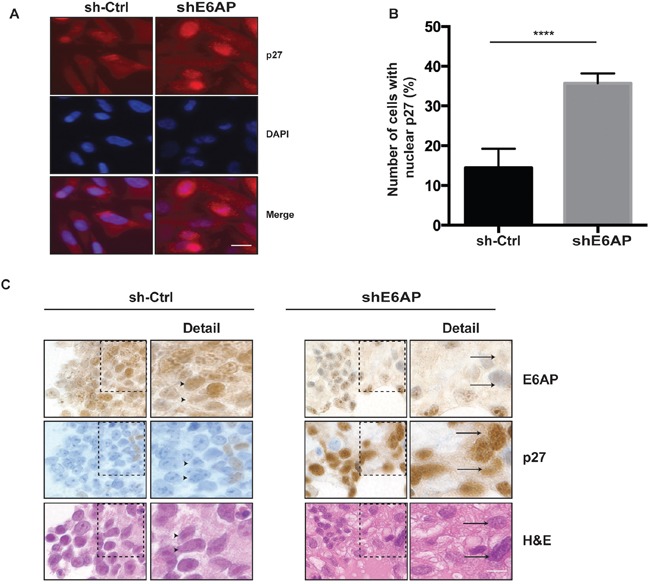
Knockdown of E6AP increases p27 nuclear localization *in vitro* and *in vivo* **(A)** PC-3 cells transduced with sh-Ctrl and shE6AP were treated with 0.2μg/mL Dox for 96hrs. Immunofluorescence analysis of cells stained for p27 and DAPI. Scale bar, 20μm. **(B)** The number of sh-Ctrl and shE6AP expressing cells with positive nuclear p27 staining was quantified. At least 400 cells per sample were scored. The results were expressed as the percentage of cells showing nuclear p27 staining. The graph represents mean ± S.D. *P-values* were calculated by unpaired Student *t*-test (****p<0.0001). **(C)** Immunohistochemistry analysis of DU145 xenografts transduced with sh-Ctrl or shE6AP treated with Dox and collected at ethical endpoint. The arrowheads indicate cells expressing high E6AP/low p27 and the arrows indicate cells expressing low E6AP/high p27 (see ‘detail’ on the right). Scale bar, 8μm.

P27 activity is not only regulated by its protein expression levels, but also by its subcellular localization [16, 17]. P27 localization is used as a marker of disease progression and therapeutic outcome in cancers of the breast, lung, ovarian, cervix, oral and lung (reviewed in [13]). We next examined whether E6AP affected p27 subcellular localization by performing immunofluorescence analysis in PC-3 cells. Upon E6AP knockdown, p27 protein primarily accumulated in the nucleus (Figure 3A, 3B) resulting in a ~20% increase in the number of cells expressing nuclear p27 compared to sh-Ctrl (Figure 3B). Further, we analysed whether knockdown of E6AP also affected p27 protein subcellular localization *in vivo*. Analysis of p27 expression by immunohistochemistry in DU145 xenografts collected at ethical endpoint as described in Figure 2C, revealed that knockdown of E6AP increased the number of cells expressing nuclear p27 compared with the sh-Ctrl (Figure 3C). Together, these results demonstrated that E6AP knockdown restored p27 protein levels resulting in an increase in nuclear p27 levels.

### E6AP regulates p27 expression at the transcriptional level

**Figure 4:**
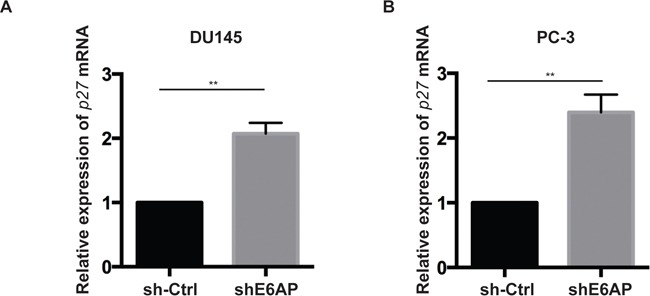
Knockdown of E6AP increases p27 transcription Quantitative RT-PCR analysis of p27 mRNA levels of sh-Ctrl and shE6AP transduced DU145 **(A)** and PC-3 **(B)** cells treated with 0.2μg/mL Dox for 96hrs. *p27* mRNA levels were normalized with *RPL37a* and expressed relative to the sh-Ctrl. Data represent the mean ± S.E.M of at least three independent experiments. *P-values* were calculated by unpaired Student *t*-test (**p<0.01).

Since E6AP acts both as an E3 ligase and a transcriptional co-activator [21, 22], we next analysed at what level(s) E6AP regulates p27 expression. We first measured the effect of E6AP on *p27* mRNA expression by quantitative real-time PCR (qRT-PCR). Knockdown of E6AP in DU145 and PC-3 cells significantly increased p27 mRNA levels compared with sh-Ctrl cells (Figure 4). The same result was observed when two independent shRNA sequences against E6AP were employed (Supplementary Figure 3) further supporting the specificity of the effect of E6AP on p27 transcription. These results support the notion that E6AP regulates p27 at the transcriptional level.

### E6AP represses p27 transcription in an E2F1-dependent manner

**Figure 5:**
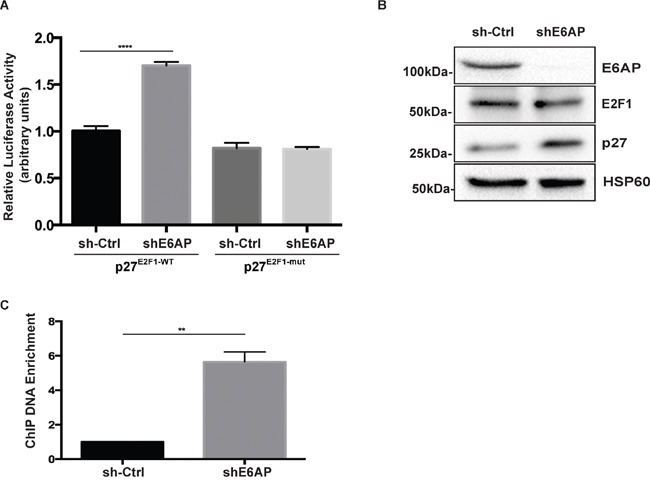
E6AP regulates p27 transcription in an E2F1-dependent manner **(A)** Analysis of p27 promoter activation by luciferase assay in PC-3 cells transduced with inducible sh-Ctrl or shE6AP and transiently transfected as indicated. Data are mean ± S.D. and are representative of three independent experiments. *P-values* were calculated by unpaired Student *t*-test (****p<0.0001). **(B)** Western blotting showing the efficiency of E6AP knockdown and the expression levels of p27, E2F1 and HSP60 (internal control) proteins. **(C)** PC-3 cells stably transduced with either sh-Ctrl or shE6AP were treated with 0.2μg/mL Dox for 96hrs before performing ChIP with an anti-E2F1 antibody. The graph represents ChIP-qPCR analysis of E2F1 binding to the p27 promoter in the presence (sh-Ctrl) or absence of E6AP (shE6AP). Data is mean ± S.E.M of three independent experiments (**p<0.01), unpaired Student *t*-test.

E2F1 is a key transcriptional activator of p27 [23]. We have recently reported that E6AP interacts with E2F1 and inhibits its transcriptional activity [24]. We therefore studied whether E6AP repressed p27 expression by inhibiting its E2F1-dependent transcription. PC-3 cells expressing inducible shE6AP or sh-Ctrl were transfected with a luciferase reporter construct driven by a p27 promoter containing an E2F1 binding site (p27^E2F1-WT^) or a deletion mutant lacking the E2F1 binding site (p27^E2F1-mut^) as a control [23]. Knockdown of E6AP increased the activation of p27^E2F1-WT^ promoter by 1.7 fold compared with the sh-Ctrl cells but did not have any effect on cells transfected with p27^E2F1-mut^ promoter (Figure 5A), suggesting that E6AP repressed p27 transcription in an E2F1-dependent manner.

To gain insight into how E6AP regulates E2F1 transcriptional activity, we tested whether E6AP promoted E2F1 protein degradation in PC cells. This possibility was ruled out as knockdown of E6AP had no significant impact on E2F1 protein levels (Figure 5B), consistent with our recent findings in lung cancer cells [24]. To test whether E6AP affected E2F1 binding to the p27 promoter, we performed a chromatin immunoprecipitation (ChIP) assay in PC-3 cells. Upon E6AP knockdown, we observed a ~6 fold increase in the amount of p27 promoter DNA precipitated with an anti-E2F1 antibody (Figure 5C). Overall, our results suggest that E6AP reduced p27 expression levels by interacting with E2F1 and diminishing binding of E2F1 to the p27 promoter, resulting in a decrease in p27 transcription.

### Double knockdown of E6AP and p27 partially restores PC cell growth

**Figure 6:**
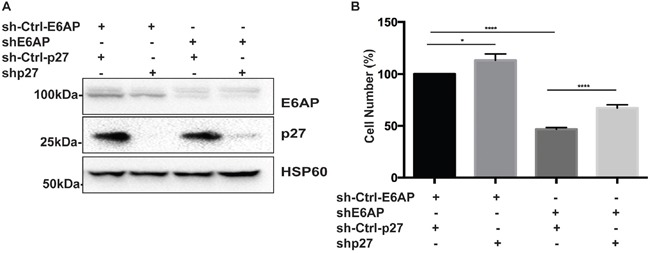
Double knockdown of E6AP and p27 partially rescues PC cell growth **(A)** Immunoblotting analysis of E6AP and p27 protein levels in PC-3 cells transduced with shRNA against E6AP and/or p27 along with hairpin control for E6AP and vector control for p27 after 96hrs of Dox treatment. **(B)** Effect of E6AP and p27 knockdown on PC-3 cell proliferation after 96hrs of Dox treatment was analyzed by cell counting. The experiments were repeated at least three times with technical triplicates. The graph represents mean ± S.E.M of three independent experiments and *P-values* were calculated by unpaired Student *t*-test (****p<0.0001, *p<0.05).

To evaluate the contribution of p27 to the overall effect of E6AP knockdown on PC cell proliferation, we compared the growth rate of PC-3 cells transduced with either a Dox-inducible shRNA against E6AP alone or together with a Dox-inducible shRNAs against p27. Upon Dox treatment for 96hrs, the expression levels of both E6AP and p27 were reduced (Figure 6A). Knockdown of E6AP reduced cell numbers by 53% compared with the sh-Ctrl (Figure 6B). We argue that this reduction in cell growth is due to growth inhibition rather than cell death since we did not detect an increase in floating dead cells in the cultures, consistent with our previous findings [9]. Importantly, concurrent knockdown of p27 together with E6AP knockdown partially restored cell growth by 20% (Figure 6B), suggesting that E6AP knockdown inhibited the growth of PC cells at least in part by restoring p27 expression. Together, our results demonstrate that E6AP is a negative regulator of p27 in PC cells, and that E6AP knockdown restored p27 levels and induced tumor suppression in these cells.

## DISCUSSION

Restoration of tumor suppression by proteasome inhibition is an attractive therapy for PC. In preclinical experiments, Bortezomib was demonstrated to induce growth arrest and apoptosis in PC cells [1, 2, 25]. Further, promising remedial responses were achieved using Bortezomib as a mono-therapy in initial clinical trials in PC patients with advanced disease [1, 26, 27], and in combination with chemotherapy in phase II clinical trials [28]. New generation proteasome inhibitors are currently in clinical development (reviewed in [29]), however their targets in PC are largely unknown. We hypothesized that rescuing tumor suppressor proteins jettisoned to the proteasome under the directive of high levels of the E3 ligase E6AP is of therapeutic relevance. In HPV-related cancers, E6AP targets the key tumor suppressor p53 for degradation [4] in an E6-dependent manner. In the context of non-HPV cancers, our previous studies demonstrated that E6AP targets PML in B-cell lymphoma [30] and PC [8, 9]. However, as argued above, the analyses of PC samples revealed a sub-population of patients (12%) with high E6AP protein levels that lacked corresponding reduction in PML expression levels. The tumor suppressor(s) targeted in these PC patients remain unknown.

In this study we analysed two independent cohorts of PC samples and identified a subset of patients expressing high E6AP and low p27 protein levels. Notably, 46% of patients of TMA1 (n=47) were identified with this profile, whereas only 26% of tumors in TMA2 (n=117) showed this profile (Figure 1). This apparent difference can potentially be explained by the different grades that each TMA represents. TMA1 is largely compromised of samples from patients with higher Gleason score (Gleason score 6-9) cancers compared with TMA2 (Gleason score 3-7). It is tempting to suggest that the inverse correlation between high E6AP and low p27 is more relevant to patients with more advanced PC. This would be consistent with downregulation of p27 (as evident in late-stage PC [13, 17, 31]), coincident with its reduced nuclear expression, as a predictor of biochemical disease recurrence, correlating with the highest Gleason grade (reviewed in [13]). Pertinently, we demonstrated that knockdown of E6AP increases the nuclear accumulation of p27 in PC cells (Figure 3). This is consistent with a correlation of high nuclear expression of E6AP and low nuclear p27 levels in PC tumors (Figure 1). We therefore propose that E6AP provides a molecular mechanism for the reduction in p27 expression in these patients.

Deregulation of p27 expression in cancer has largely been attributed to proteasomal degradation (reviewed in [13]), while its deregulation at the transcription level is still to be explored. Here we showed that knockdown of E6AP increases p27 mRNA levels (Figure 4) by inhibiting E2F1-mediated transcription of p27 (Figure 5). This is consistent with our recent finding that E6AP inhibits E2F1 transcriptional activity in a pRb-independent manner [24]. It is likely that in addition to the effect of E6AP on p27 transcription, E6AP also affects p27 at the protein level, as previously demonstrated in neuronal cells [20].

Our study provides a proof of concept that targeting E6AP, in our case by knockdown, is sufficient to partially restore p27 nuclear expression *in vitro* and *in vivo*. Countering this, restoration of p27 expression partially rescued the cell growth inhibition induced by E6AP knockdown (Figure 6). This demonstrates the important link to PC of elevated E6AP expression and reduced levels of p27. Thus, targeting E6AP is a potential therapeutic strategy to restore p27-mediated tumor suppression in high grade and high stage PC.

## MATERIALS AND METHODS

### Tissue microarray (TMA) analysis

TMA1 was constructed from PC biopsies collected from the Urology Department at Regina Elena National Cancer Institute, Rome, Italy. These biopsies were the archived samples of patients who underwent prostatectomy without any pharmacological treatment. TMA2 was constructed from PC biopsies from archived radical prostatectomy specimens that were collected from the Royal Berkshire Hospital, Reading, United Kingdom [32]. TMA 1 and 2 were stained using E6AP and p27 antibodies. Samples were scored for both the intensity of staining and the proportion of cells stained. Intensity of nuclear staining was scored as 0 (absent), 1 (low), 2 (intermediate) or 3 (strong), and the proportion of tumor cells stained was then categorised relative to percentage staining 0, 1<=25%, 2=>25-50%, 3=>50-75% and 4=>75%. The histoscore was calculated as intensity*categorised proportion of stained tumor cells to give a score between 0-12.

### Cell culture

DU145 and PC-3 cells (ATCC, Manassas, VA, USA) were maintained in DMEM (Gibco, Grand Island, NY, USA), supplemented with 10% fetal bovine serum (Gibco) and 0.1% Penicillin/Streptomycin (Sigma, St Louis, MO, USA). Cells were incubated in a humidified incubator at 37°C with 5% CO2. For passaging, cells were washed with PBS-EDTA and trypsinized with 0.25% trypsin (Gibco). Trypsin was neutralized using DMEM with 10% fetal bovine serum. Trypsinized cells were subsequently centrifuged at 1000 rpm for 5 min at 4°C, and the resultant pellet was suspended in media for sub-culturing. Cell counts were performed using a haemocytometer.

### Xenograft studies

For the tumor cell line xenograft experiments, the experiments were performed as explained in [9].

### Plasmids transfection and viral production

Lentivirus expressing shRNA were previously described in [33]. The sequences used were: shE6AP (Forward 5′- CGCGTCCCCGAAGCAGTTGTATGTGGA ATT CAAGAGATTCAAGAGATTCCACATACAACTGCTTCTTTTTGGAAAT-3′ and reverse 5′-CGATTTCCAAAAAGAAGCAGTTGTATGTGGAATCTCTTGAAT CTCTTG AATTCCACATACAACTGCTTCGGGGAG CG-3′), sh-Ctrl (Forward 5′- TCCC GAAGCAGTATGATGTGGAATTCAAGAGATTCCACATCATACTGCTTC TTTTTC-3′ and reverse 5′-TCGAGAAAAAGAAGCA GTATGATGTGGAATCTCTTGAATTCC ACATCATAC TGCTTC -3′), shp27 (Forward 5′-TCCCCGACGATTCT TCTAC TCAATTCAAGAGATTGAGTAGAAGAATCGTCGTTTTTC-3′ and reverse 5′-TCGA GAAAAACGACGATTCTTCTACTCAATCTCTGAATTGAGTAGAAGAA TCGTCG-3′) and empty vector control for shRNA against p27. Viral production and cells infection was performed as previously described [30]. shRNAs expression was induced by treating DU145 and PC-3 cells with 0.2μg/ml Dox (Sigma) for the indicated periods.

### Western blotting

DU145 and PC-3 cells were seeded and treated with Dox for 96hrs (Dox was topped up every 48hrs). Immunoblotting was performed as previously described [24]. Antibodies used were anti-E6AP (E6AP-330, Sigma), anti-p27 (BD bioscience, San Jose, CA, USA), anti-HSP60 (H-300, Santa-Cruz, Dallas, TX, USA) and anti-E2F1 (sc-193, Santa Cruz). The secondary antibodies used were goat anti-rabbit IgG or anti-mouse IgG (Life Technologies, Pleasanton, CA, USA).

### Immunohistochemistry

Immunohistochemistry was performed as described in [9]. Antibodies used were anti-E6AP (Serotec, Oxford, UK) and anti-p27 (BD biosciences).

### Quantitative real-time PCR (qRT-PCR)

Cells were collected and RNA was isolated using TRIzol (Life Technologies) as per the manufacturer's protocol. cDNA was synthesized from the RNA using M-MLV reverse transcription kit (Promega, Madison, WI, USA). Primers used: human p27 (forward 5′-GGCCTCAGAAGACGTCAAAC-3′ and reverse 5′-ACAGGATGTCCATTCCATGA-3′); human RPL37a (internal control) forward (5′-GCCAGCACGC CAAGTACAC-3′ and reverse 5′-CCCCACAGCTC GTCTCTTCA-3′). PCR was performed on StepOnePlus PCR machine (Applied Biosystems, Foster City, CA, USA) using Fast SYBR Green Master Mix (Applied Biosystems). Gene expression was calculated using the relative C_t_ method. All the assays were performed in technical triplicates in at least three independent experiments.

### Immunofluorescence assay

Immunofluorescence assay was performed as explained in [12]. Photographs were taken using BX-51 microscope (Olympus). The exposure time was kept constant all throughout the experiment. A minimum of 400 cells were scored for p27 nuclear intensity in both sh-Ctrl and shE6AP transduced cells. The results were expressed as the percentage of cells showing nuclear p27 staining. The experiments were performed three independent times with technical duplicates.

### Luciferase assay

PC-3 cells expressing inducible shE6AP or sh-Ctrl were transfected with p27 promoter containing an E2F1 binding site (p27^E2F1-WT^) or a deletion mutant lacking the E2F1 binding site (p27^E2F1-mut^) as a control (provided by Prof. Jiandong Chen) and Renilla luciferase construct as the internal control. Luciferase assay was performed as described in [24].

### Chromatin immunoprecipitation (ChIP) assay

ChIP assay was performed as described previously [24] using anti-rabbit E2F1 (sc-193, Santa Cruz) or rabbit IgG control (sc-2027, Santa Cruz) and protein G-coupled magnetic beads (Dynabeads Protein G, Invitrogen, Carlsbad, CA, USA). DNA samples were extracted using phenol/chloroform/isoamyl alcohol (Life Technologies, Carlsbad, CA, USA) and precipitated with sodium acetate. Quantitative real-time PCR reactions were done using 1μl of immunoprecipitated material or total input using 1μM of E2F1 primers (Forward 5′- GGCCTCCCCCGCAGACCAC-3′ and reverse 5′- GTTCCGCCACCTCCCCTCGTTCC-3′)

## SUPPLEMENTARY FIGURES



## Abbreviations

PC: Prostate cancer
E6AP: E6-associated protein
CDK: cyclin dependent kinase
Dox: Doxycycline
TMA: Tissue Microarray
sh-Ctrl: short hairpin ribonucleic acid- control
C_t_: Cycle Threshold
