# Supplementary material for: E6AP promotes prostate cancer by reducing p27 expression
Source: Oncotarget. 2017 Apr 19;8(26):42939–48. doi: 10.18632/oncotarget.17224 (PMC5522117; doi:10.18632/oncotarget.17224)
Supplement: Supplementary file 1 [file oncotarget-08-42939-s001.pdf]

E6AP promotes prostate cancer by reducing p27 expression

SUPPLEMENTARY FIGURES

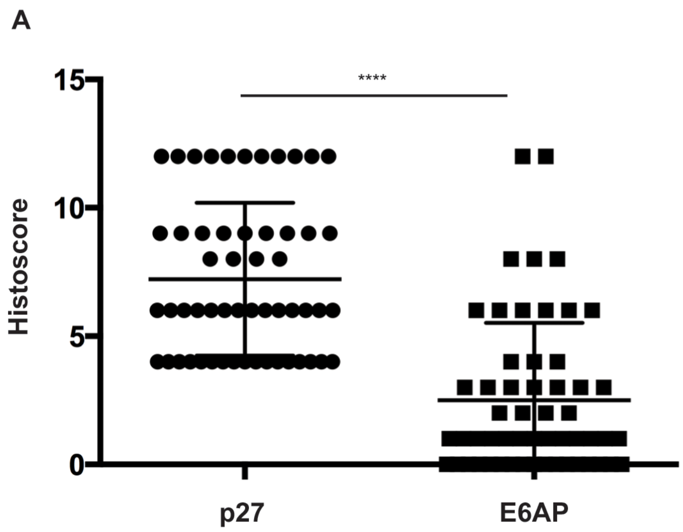

Supplementary Figure 1: Expression levels of E6AP and p27 inversely correlate in PC. TMA2 samples from PC patients were stained for E6AP and p27 and scored both for the proportion of stained cells and the staining intensity. Intensity of staining ranged from 0 to 3 and the proportion of cells stained was based on a scale of 0 to 4 (0 to >80% respectively). The product of the intensity in the nucleus and the categorized proportion of cells stained was plotted as histoscore with S.D. *P-values* were calculated by unpaired student *t*-test (****$p<0.0001$).

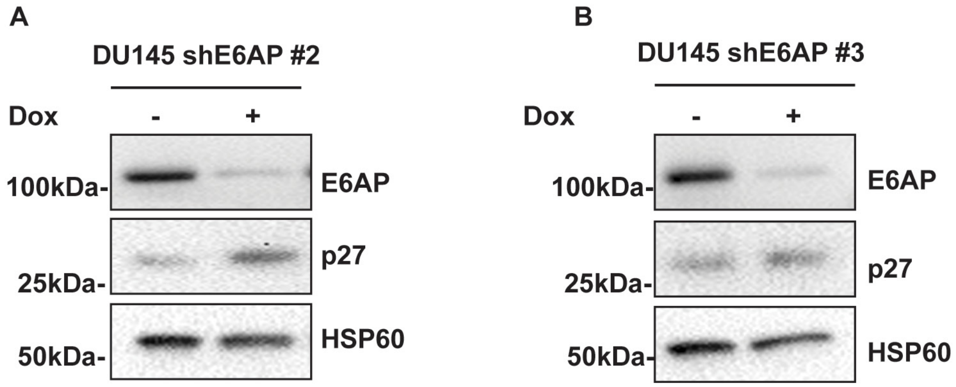

Supplementary Figure 2: Knockdown of E6AP restores p27 protein expression in DU145 cells. Cells transduced with 2 independent shRNAs sequences against E6AP were treated with 0.2µg/mL Dox for 96hrs and samples were analyzed for the expression of E6AP, p27 and HSP60 (loading control) by western blotting. Representative images of independent experiments are presented here.

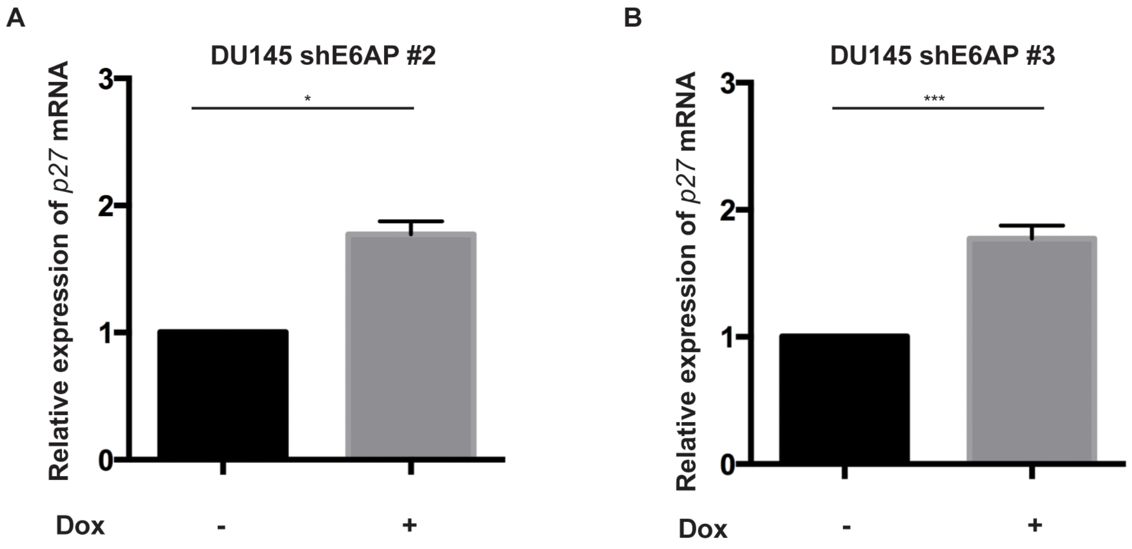

Supplementary Figure 3: Analysis of p27 mRNA levels upon E6AP knockdown using 2 different shE6AP sequences. Cells transduced with 2 different shRNAs sequences against E6AP were treated with 0.2µg/mL Dox for 96hrs and *p27* mRNA levels expression was analyzed by qPCR. *p27* levels were normalized with *RPL37a* (internal control) and expressed relative to the sh-Ctrl. The graphs represent mean ± S.D. *P-values* were calculated by unpaired student *t*-test (*$*p<0.05$; $***p<0.001$*).
